# A Span-Prior-Guided Explainable Multimodal Neural Network Method for Final-State Quality Inspection of Hairpin Windings

**DOI:** 10.3390/s26154857

**Published:** 2026-08-01

**Authors:** Xiaopeng Chang, Bangcheng Zhang, Zhi Gao, Siyu Chen, Jingru Liu

**Affiliations:** 1 School of Mechatronic Engineering, Changchun University of Technology, Changchun 130012, China; 1202201011@stu.ccut.edu.cn (X.C.); gaozhi@ccut.edu.cn (Z.G.); 1202301013@stu.ccut.edu.cn (S.C.); 2 School of Mechanical and Electrical Engineering, Changchun Institute of Technology, Changchun 130103, China; 3 FAW Tool & Die Manufacturing Co., Ltd., No. 1999, Jieda Road, Changchun 130011, China; liuqr_td@faw.com.cn

**Keywords:** hairpin windings, final-state quality inspection, span prior, explainable multimodal neural network, multimodal sensing fusion

## Abstract

For final-state quality inspection of three-dimensional stamped hairpin windings, existing studies still lack multimodal methods that integrate mechanical geometric constraints, prior-guided fusion, and decision interpretability. This study proposes a span-prior-guided explainable multimodal neural network method and develops SPIMA-Net. The final-state images were acquired at a fixed inspection station with a fixed camera position and imaging angle under a CCD vision light source. Final-state images are used as visual inputs, while geometric priors are constructed from span measurements and model-type information. A visual branch and a span branch extract image and prior features, and a span-prior-assisted gating mechanism modulates visual features to enable collaborative fusion. Experimental results show that SPIMA-Net achieves an accuracy of 98.14%, an F1-score of 96.55%, and an AUC of 0.9983 on the test set. Its nonconforming-class F1-score is improved by 10.44, 3.04, 6.27, 1.38, and 0.66 percentage points over the image-only, span-only, direct-fusion, SE-fusion, and CBAM-fusion models, respectively, while the total number of misclassifications decreases to five. Interpretability analysis shows that the model mainly focuses on span openings, end profiles, and local abnormal regions. Relative and absolute span deviations are identified as the main mechanical geometric factors affecting final-state quality classification. The proposed method provides a neuro-mechanical fusion approach that demonstrates high discriminative performance and engineering interpretability for hairpin winding quality inspection on the investigated industrial dataset.

## 1. Introduction

With the development of intelligent manufacturing and industrial quality inspection, quality assessment of complex manufactured components is shifting from experience-based inspection to intelligent inspection that integrates visual images, geometric dimensions, and structured information. For mechanically formed components, final-state quality depends not only on external morphology but also on whether key geometric dimensions satisfy design requirements. In such inspection tasks, effective use of image information requires digital image representation, visual feature extraction, and convolutional representation learning to support morphology recognition and discriminative feature extraction [1,2,3]. Therefore, combining deep visual representation with mechanical geometric priors is important for final-state quality inspection with strong discriminative capability, explicit geometric constraints, and engineering interpretability.

Hairpin windings for flat-wire motors have advantages such as high slot fill factor, high power density, compact end-winding structure, and favorable heat dissipation, making them important for traction motor stators in new energy vehicles. Zou et al. reported that winding design factors such as slot–pole combinations, conductor transposition, end connections, and parallel branches affect the manufacturability and performance stability of hairpin windings [4]. Fleischer et al. further pointed out that electric drive manufacturing involves complex process chains, where manufacturing quality is critical for large-scale electric vehicle production [5]. After three-dimensional stamping, the final opening morphology, end profile, and span dimension jointly determine the assembly compatibility and quality state of hairpin windings. Existing studies have mainly addressed forming control, welding quality monitoring, visual inspection, and process quality assurance [6,7,8,9,10], but the joint evaluation of final-state images and key span dimensions after stamping remains insufficiently studied.

Deep visual neural networks have been widely used in industrial defect recognition, anomaly detection, and assembly quality assessment. Bono et al. further emphasized that quality control in automated production lines remains challenging under highly inconsistent operating conditions [11]. Related studies have explored industrial visual anomaly detection, synthetic data-based assembly inspection, few-shot defect detection, electronic board inspection, multiview inspection, and AI-enabled product defect detection [12,13,14,15,16,17]. These studies show that visual models are effective in extracting contour, edge, and local morphological features. However, for final-state inspection tasks with explicit geometric constraints, image information alone may be affected by visually similar appearances and may not fully identify quality abnormalities caused by key geometric deviations.

For three-dimensional stamped hairpin windings, final-state images provide information on end profiles, opening regions, and local morphological abnormalities, while span-related geometric dimensions provide mechanical constraints directly associated with quality criteria. These two types of information are complementary: image-only models may ignore explicit span deviation information, whereas span-only models cannot fully capture local contour abnormalities, end bending, or asymmetric deformation. Therefore, final-state images and span-related geometric information should be jointly modeled for quality classification.

Multimodal learning provides a useful basis for such joint modeling. Baltrušaitis et al. summarized representation, alignment, fusion, and co-learning issues in multimodal machine learning [18], while Zhao et al. reviewed deep multimodal data fusion methods and highlighted the value of multisource information fusion for improving model representation and robustness [19]. Jiao et al. further categorized multimodal fusion strategies into early fusion, deep fusion, late fusion, and hybrid fusion [20]. In industrial scenarios, multimodal modeling of images, sensors, process parameters, and structured data has been applied to diagnosis, process monitoring, quality assessment, and intelligent manufacturing systems [21,22,23,24]. However, many existing multimodal models still rely on feature concatenation or conventional fusion, and span information is rarely used to actively guide the visual branch toward opening regions, end profiles, and local abnormal locations.

Attention mechanisms, gating mechanisms, and feature recalibration methods have been widely used to help neural networks focus on task-relevant regions and discriminative features. Schlemper et al. introduced attention gating to guide networks toward important regions [25], and Hu et al. proposed channel-wise feature recalibration to enhance discriminative representation [26]. These studies provide a methodological basis for prior-guided neural fusion. However, in final-state quality inspection of three-dimensional stamped hairpin windings, how to transform measured final span, theoretical span, span deviations, and model-type information into mechanical priors for visual feature modulation remains insufficiently investigated. In addition, industrial intelligent inspection models require not only classification accuracy but also engineering interpretability. Selvaraju et al. proposed Grad-CAM to visualize key image regions involved in convolutional neural network decisions [27]. Related studies in process quality management, visual quality control, industrial fault diagnosis, quality state monitoring, and zero-defect manufacturing have shown that explainable artificial intelligence can improve the transparency and practical value of industrial AI systems [28,29,30,31,32,33,34]. Therefore, final-state quality inspection of hairpin windings should not only output conforming/nonconforming decisions but also explain the image regions and geometric factors influencing the decisions.

In summary, previous studies on hairpin windings have mainly focused on forming control, welding monitoring, and visual inspection, while final-state quality inspection after three-dimensional stamping remains insufficiently studied. In this study, the final-state images were acquired at a fixed inspection station with a fixed camera position and imaging angle under a CCD vision light source. This task requires the joint evaluation of final-state morphology and span dimensions. Image-only methods cannot explicitly use span deviation information, whereas span-only methods cannot capture local contour abnormalities. Conventional multimodal fusion usually relies on feature concatenation and lacks active guidance from span priors to visual features. Therefore, a span-prior-guided and interpretable multimodal method is needed for final-state quality inspection of three-dimensional stamped hairpin windings.

To address these issues, this study proposes a span-prior-guided explainable multimodal neural network method for final-state quality inspection of hairpin windings and constructs the Span-Prior-Informed Multimodal Assessment Network (SPIMA-Net) as its core implementation model. The proposed method integrates final-state image representations with span-related geometric priors. Through a visual branch, a span branch, and a prior-assisted gating mechanism, it enables collaborative fusion between neural visual features and mechanical geometric constraints, and is applied to final-state quality classification and risk scoring of three-dimensional stamped hairpin windings for flat-wire motors.

The main contributions of this study are summarized as follows:

(1) A multisource collaborative representation method is proposed for final-state quality inspection of hairpin windings.

Final-state image information and mechanical span geometric priors are jointly modeled, enabling the model to simultaneously exploit visual morphological features and span deviation information for final-state quality classification and risk quantification.

(2) A span-prior-guided multimodal neural network model, SPIMA-Net, is constructed.

Mechanical geometric priors are encoded through a span branch, and a span-prior-assisted gating mechanism is designed to modulate visual features, thereby enhancing the model’s discriminative ability for span opening regions, end profiles, and local abnormal regions.

(3) A dual-sided explainable analysis method is proposed for image regions and mechanical span factors.

By combining Grad-CAM with permutation feature importance analysis, the key abnormal regions attended to by the model are identified from the image side, while the contributions of major geometric factors to quality classification are quantified from the span side. This result supports the interpretability and practical applicability of the proposed method for final-state quality inspection of hairpin windings.

## 2. Methods

### 2.1. Problem Definition and Span Prior Construction

**Figure 1:**
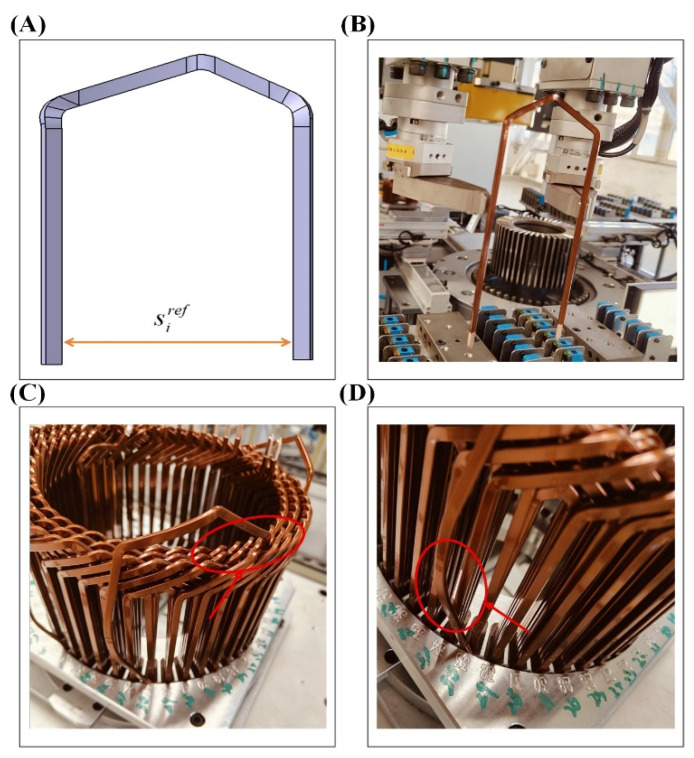
Structure and assembly of the stamped hairpin winding: (**A**) simplified structure and definition of the theoretical span; (**B**) practical assembly scene; (**C**,**D**) examples of local interference caused by excessive span deviation.

The final-state quality of three-dimensional stamped hairpin windings is jointly affected by end morphology, span dimensions, and differences among model types. Figure 1A presents the simplified structure of the stamped hairpin winding and the definition of the theoretical span. The stamped hairpin winding is formed from a flat copper conductor through bending and three-dimensional stamping, where the theoretical span $s_{i}^{ref}$ denotes the designed distance between the two end regions. Figure 1B shows the practical assembly scene of the hairpin winding, in which the final-state span directly affects its insertion accuracy and assembly compatibility. When the span deviation is excessive, assembly difficulty or local interference may occur, as shown in Figure 1C,D. Therefore, final-state quality inspection should consider both visual morphology information and span-related geometric information.

Based on the stamped hairpin winding structure and span definition, to enable joint modeling of final-state image information and mechanical geometric priors, the i-th sample is defined as:

(1) $$x_{i}=\left\{I_{i},s_{i},s_{i}^{ref},m_{i},y_{i}\right\}$$

where $I_{i}$ denotes the final-state image of the i-th sample, $s_{i}$ denotes the measured final span, $s_{i}^{ref}$ denotes the theoretical span corresponding to the model type, $m_{i}$ denotes the model-type information, and $y_{i}\in \left\{0,\;1\right\}$ denotes the quality label, where 1 indicates conforming and 0 indicates nonconforming.

Because the theoretical span varies across different hairpin winding model types, using only the measured final span $s_{i}$ cannot accurately reflect its deviation from the target dimension. Therefore, the absolute span deviation is defined as:

(2) $$e_{i}=\left|s_{i}-s_{i}^{ref}\right|$$

and the relative span deviation is further defined as:

(3) $$d_{i}=\frac{\left|s_{i}-s_{i}^{ref}\right|}{s_{i}^{ref}+\varepsilon }$$

where ε is a small constant introduced to avoid division by zero. In this study, ε was fixed as 1 × 10^−8^. This value was used only for numerical stability and was not tuned according to different model types. The absolute span deviation $e_{i}$ represents the actual dimensional deviation, while the relative span deviation $d_{i}$ is used to reduce the influence of scale differences in theoretical span among different model types on quality classification.

Based on these definitions, the span prior vector is constructed as:

(4) $$g_{i}=\left[s_{i},s_{i}^{ref},e_{i},d_{i},m_{i}\right]$$

where the model-type information $m_{i}$ can be encoded using one-hot encoding. This vector provides mechanical geometric information, including the measured dimension, target dimension, deviation magnitude, and model-type context. The variables $s_{i}$, $s_{i}^{ref}$, $e_{i}$, and $d_{i}$ in the vector are not independent raw observations but correlated geometric descriptors constructed for final-state span quality judgment. Specifically, $e_{i}$ and $d_{i}$ characterize the deviation of $s_{i}$ from $s_{i}^{ref}$ from the perspective of absolute span deviation and relative span deviation, respectively. Therefore, this interdependence does not introduce an additional modeling problem but helps explicitly represent the engineering judgment information of span deviation and provides structured geometric priors for subsequent multimodal feature fusion.

The objective of this study is to learn the following mapping:

(5) $$f:\left\{I_{i},g_{i}\right\}\to \left\{{\hat{y}}_{i},{\hat{r}}_{i}\right\}$$

where ${\hat{y}}_{i}$ denotes the predicted quality class, and ${\hat{r}}_{i}$ denotes the risk score. Since this study focuses on identifying nonconforming samples, the predicted probability of the nonconforming class is defined as the risk score. A higher risk score indicates a greater risk of final-state quality abnormality.

### 2.2. SPIMA-Net Network Architecture

**Figure 2:**
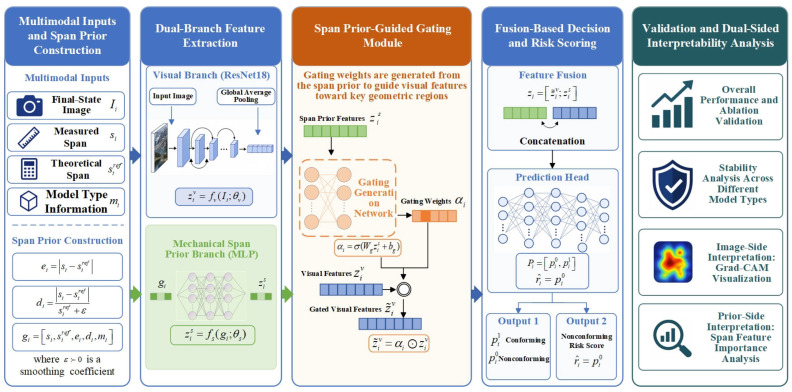
SPIMA-Net span-prior-guided multimodal final-state quality inspection framework.

As shown in Figure 2, SPIMA-Net is constructed as a span-prior-guided multimodal classification framework. The model takes the final-state image $I_{i}$ and the span prior vector $g_{i}$ as two complementary inputs. The visual branch maps $I_{i}$ into image feature representation $z_{i}^{v}$, while the span branch encodes $g_{i}$ into span prior feature representation $z_{i}^{s}$. Unlike direct feature concatenation, SPIMA-Net uses the span prior representation to generate gating weights, which are then applied to the visual features for adaptive feature modulation. This design allows the geometric prior information to participate in the visual feature learning process before multimodal fusion. The modulated visual representation and span prior representation are subsequently concatenated and passed into the classification head to obtain the conforming/nonconforming prediction and the nonconforming risk score.

In the visual branch, ResNet18 is adopted as the visual backbone for extracting final-state image features [35]. ResNet18 is a residual convolutional network composed of stacked residual blocks with shortcut connections, which helps stable extraction of contour, edge, and local morphology features from final-state images and reduces overfitting risk under limited industrial samples:

(6) $$z_{i}^{v}=f_{v}\left(I_{i};\theta_{v}\right)$$

where $z_{i}^{v}$ denotes the visual feature vector, $\theta_{v}$ represents the parameters of the visual branch, and $f_{v}(•)$ denotes the visual feature extraction function.

In the span branch, the span prior vector $g_{i}$ constructed in Section 2.1 is fed into a multilayer perceptron (MLP) encoder to obtain the span prior feature:

(7) $$z_{i}^{s}=f_{s}\left(g_{i};\theta_{s}\right)$$

where $z_{i}^{s}$ denotes the span prior feature vector, while $f_{s}(•)$ and $\theta_{s}$ represent the span branch encoding function and its parameters, respectively. The span branch adopts a two-layer fully connected structure, with ReLU activation and Dropout introduced to suppress overfitting.

To avoid passive concatenation of image features and span information only at the final stage, this study designs a span-prior-guided gating mechanism. This module uses span prior features to generate gating weights with the same dimensionality as the visual features:

(8) $$\alpha_{i}=\sigma \left(W_{g}z_{i}^{s}+b_{g}\right)$$

where $\alpha_{i}$ denotes the gating weight generated from the span prior, $\sigma \left(•\right)$ is the sigmoid function, and $W_{g}$ and $b_{g}$ are the weight matrix and bias term of the gating mapping, respectively. In this study, the visual feature vector extracted by the ResNet18 branch is $z_{i}^{v}\in R^{512}$, and the span prior feature vector is $z_{i}^{s}\in R^{32}$. The linear gating layer maps $z_{i}^{s}$ to $\alpha_{i}\in R^{512}$, which has the same dimension as $z_{i}^{v}$. Therefore, the visual features are modulated by element-wise multiplication as follows:

(9) $${\tilde{z}}_{i}^{v}=\alpha_{i}⊙z_{i}^{v}$$

where ⊙ denotes element-wise multiplication, and ${\tilde{z}}_{i}^{v}$ denotes the visual feature representation modulated by the span prior. This mechanism enables span prior information to participate in visual feature modulation and strengthens the discriminative representation of the model.

The modulated visual features are then fused with the span prior features:

(10) $$z_{i}=\left[{\tilde{z}}_{i}^{v};z_{i}^{s}\right]$$

where $z_{i}$ denotes the fused multimodal feature vector, and $\left[{\tilde{z}}_{i}^{v};z_{i}^{s}\right]$ denotes the concatenation of the span-prior-modulated visual feature and the span prior feature.

The class probabilities are then produced by a fully connected classification head:

(11) $$P_{i}=\mathrm{soft}\max\left(W_{2}\phi \left(W_{1}z_{i}+b_{1}\right)+b_{2}\right)$$

where $W_{1}$, $W_{2}$, $b_{1}$ and $b_{2}$ are the parameters of the classification head, and $\phi \left(•\right)$ denotes a nonlinear activation function. The fully connected layers in the span-related branch and the fusion classification head use ReLU activation. The gating module adopts a sigmoid activation function to generate element-wise modulation weights for visual features, and the final classification logits are converted into class probabilities using the softmax function. The Dropout rate is set to 0.2 in the span-related branch and 0.3 in the fusion classification head.

(12) $$P_{i}=\left[p_{i}^{0},p_{i}^{1}\right]$$

where $P_{i}^{0}$ and $P_{i}^{1}$ denote the softmax outputs corresponding to the nonconforming and conforming classes, respectively. The risk score is defined as:

(13) $${\hat{r}}_{i}=p_{i}^{0}$$

That is, a higher softmax output for the nonconforming class indicates a higher final-state instability risk. In this study, ${\hat{r}}_{i}$ is used to represent the relative risk degree that the model assigns a sample to the nonconforming class. Considering that softmax outputs may be affected by data distribution bias and class imbalance, it is not interpreted as an absolute probability estimate after additional probability calibration.

In summary, SPIMA-Net does not simply concatenate image information and span information. Instead, it employs a span-prior-guided gating mechanism that enables mechanical geometric priors to participate in visual feature modulation, thereby achieving joint output of final-state quality classification and risk scoring.

### 2.3. Comparative Models and Ablation Experiment Design

To validate the effectiveness of the proposed SPIMA-Net and analyze the roles of image information, span priors, multimodal fusion, and the gating mechanism in final-state quality inspection, six comparative models are constructed: the image-only model, the span-only model, the direct-fusion model, the SE-fusion model, the CBAM-fusion model, and SPIMA-Net. The structural components and input information of these six models are summarized in Table 1.

Among them, the image-only model takes final-state images as input and uses ResNet18 for quality classification, thereby evaluating the independent discriminative capability of visual morphological information. The span-only model takes the span prior vector $g_{i}$ as input and adopts an MLP for classification, thereby assessing the contribution of mechanical geometric priors to quality decision-making. The direct-fusion model simultaneously introduces image features and span features, but performs multimodal fusion only through feature concatenation, so as to evaluate the effect of conventional multimodal fusion. The SE-fusion and CBAM-fusion models introduce attention-based fusion modules to evaluate whether general attention-based mechanisms can improve multimodal feature interaction compared with direct feature concatenation. SPIMA-Net further incorporates a span-prior-guided gating mechanism on the basis of direct fusion, thereby examining the effectiveness of mechanical geometric priors in modulating visual features. Therefore, this set of experiments is used to analyze the effects of image information, span priors, direct fusion, attention-based fusion, and span-prior-guided gating on final-state quality classification performance.

### 2.4. Joint Loss and Dual-Sided Interpretability Analysis

To jointly ensure class discriminative and span-consistency constraints for risk scoring, this study adopts a joint optimization scheme that combines classification loss with a span auxiliary loss.

The classification loss is defined using weighted cross-entropy:

(14) $$L_{cls}=-\frac{1}{N}\sum_{i=1}^{N}\left[\alpha_{1}y_{i}\log p_{i}^{1}+\alpha_{0}\left(1-y_{i}\right)\log p_{i}^{0}\right]$$

where N denotes the number of samples, while $\alpha_{1}$ and $\alpha_{0}$ are the class weights for the conforming and nonconforming classes, respectively, which are used to mitigate the influence of class imbalance during training.

To make the risk score reflect the degree of final-state span instability, a span auxiliary loss is further introduced. Considering that the risk score ${\hat{r}}_{i}$ lies within [0, 1], the relative span deviation $d_{i}$ is normalized by the maximum value in the training set as follows:

(15) $${\tilde{d}}_{i}=\frac{d_{i}}{d_{\max}^{train}}$$

and clipped to the interval [0, 1]. Subsequently, ${\tilde{d}}_{i}$ is used as the risk constraint target, and the span auxiliary loss is defined as:

(16) $$L_{span}=\frac{1}{N}{\sum_{i=1}^{N}\left({\hat{r}}_{i}-{\tilde{d}}_{i}\right)}^{2}$$

Therefore, the total loss function is defined as:

(17) $$L=L_{cls}+\lambda L_{span}$$

where λ is a balancing coefficient, which was set to 0.20 in this study. The class weights in the weighted cross-entropy loss were calculated according to the inverse class frequency in the training set. Since the nonconforming class was labeled as 0 and the conforming class as 1, the class weights were set to α_0_ = 1.8646 for the nonconforming class and α_1_ = 0.6832 for the conforming class. This joint loss enables the model to perform conforming/nonconforming classification while producing risk scores that are consistent with the degree of span deviation.

For interpretability analysis, this study reveals the model decision basis from both the image side and the span side. On the image side, Grad-CAM is used to generate class activation heatmaps for locating the key abnormal regions attended to by the model. On the span side, permutation feature importance analysis is performed for the measured final span, theoretical span, absolute span deviation, relative span deviation, and model-type information. Each factor is perturbed separately. The changes in F1-score and AUC are then used to quantify its contribution to quality classification.

In summary, the proposed framework combines joint-loss optimization for class decision and span-risk constraint with dual-sided interpretation from image regions and span-related factors.

## 3. Results

### 3.1. Dataset and Experimental Settings

To validate the effectiveness of the proposed SPIMA-Net for final-state quality inspection of hairpin windings, this study uses field-collected samples of flat-wire motor hairpin windings after the three-dimensional stamping process. The samples were collected from one stator production batch, which contained seven different hairpin winding geometries/model types. Image acquisition was performed at a fixed inspection station using one industrial camera, GV-CRM120-10, under a CCD vision light source. During acquisition, the camera position, imaging angle, and lighting condition were kept fixed.

The dataset consists of final-state images, quality labels, measured final spans, theoretical spans, span deviation features, and model-type information. The dataset was divided into training and test sets using a stratified 80%/20% random split according to the quality labels and model types. The same image or the same physical sample was not duplicated in both the training and test sets. Therefore, the experimental results were obtained under a sample-level random partition within the investigated stator production batch. The dataset composition and partitioning are summarized in Table 2.

In the experiments, nonconforming samples are treated as the positive class, with emphasis placed on evaluating the model’s ability to identify final-state quality abnormalities. The evaluation metrics include accuracy, precision, recall, F1-score, and AUC. Accuracy measures the overall classification correctness; precision and recall reflect the correctness and completeness of nonconforming sample identification, respectively; F1-score provides a comprehensive evaluation of nonconforming-class recognition performance; and AUC assesses the overall separability between conforming and nonconforming samples.

Following the comparative design in Section 2.3, the compared models were trained and evaluated under the same training–test split. The network input image size was set to 224 × 224. During training, light image augmentation was applied, including brightness and contrast perturbation with a factor of 0.05, while no augmentation was used during testing. Images were normalized using the ImageNet mean and standard deviation.

**Figure 3:**
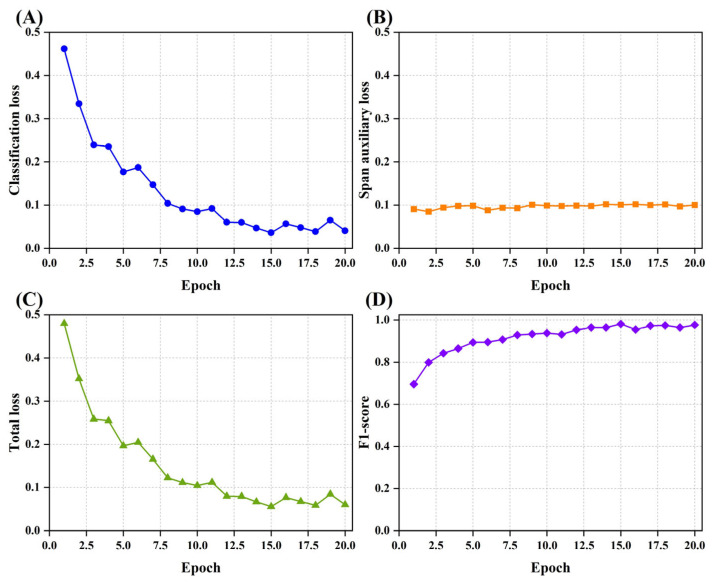
Training convergence curves of SPIMA-Net over 20 epochs. (**A**) Classification loss; (**B**) span auxiliary loss; (**C**) total loss; (**D**) F1-score.

Span-related variables were standardized using the mean and standard deviation calculated from the training set. For the span auxiliary loss, the relative span deviation was normalized by the maximum relative span deviation in the training set and clipped to [0, 1]. ResNet18 was trained from scratch. AdamW was used with a learning rate of 1 × 10^−4^, a weight decay of 1 × 10^−4^, a batch size of 16, and 20 training epochs. The balancing coefficient in the total loss was set to λ=0.20, and the weighted cross-entropy class weights were set to $\alpha_{0}=1.8646$ for the nonconforming class and $\alpha_{1}=0.6832$ for the conforming class. The main random seed was 42. No additional learning-rate scheduler or early stopping strategy was used; all models were trained under this fixed 20-epoch setting. The experiments were implemented in Python 3.10.7 using PyTorch 2.13.0 and run in the PyCharm 2023.1 environment on a computer equipped with an NVIDIA GeForce RTX 2060 (6 GB) GPU. On this platform, SPIMA-Net has 11.27 M parameters and achieved an average inference time of 166.24 ms/sample, corresponding to 6.02 FPS. To verify the sufficiency of the 20-epoch training schedule, the training convergence curves of SPIMA-Net were further analyzed, as shown in Figure 3A–D. The classification loss and total loss gradually decreased and stabilized within 20 epochs, while the span auxiliary loss remained at a low and stable level. Meanwhile, the F1-score increased and became stable in the later epochs, indicating that the model had basically converged under the adopted training setting. The best model was selected mainly according to the F1-score of the nonconforming class. Overfitting was controlled using light augmentation, Dropout, weight decay, and class-weighted cross-entropy loss.

### 3.2. Comparative Model and Ablation Analysis

To validate the effectiveness of the proposed SPIMA-Net and analyze the roles of image information, span priors, and the prior-guided gating mechanism in final-state quality classification, six models are compared: the image-only model, the span-only model, the direct-fusion model, the SE-fusion model, the CBAM-fusion model, and SPIMA-Net. Their overall performance on the test set is reported in Table 3.

In this study, nonconforming samples are treated as the positive class. Accordingly, TP denotes the number of truly nonconforming samples correctly predicted as nonconforming, FN denotes the number of truly nonconforming samples incorrectly predicted as conforming, FP denotes the number of truly conforming samples incorrectly predicted as nonconforming, and TN denotes the number of truly conforming samples correctly predicted as conforming. The evaluation metrics are calculated as follows:

(18) $$Accuracy=\frac{TP+TN}{TP+TN+FP+FN}$$

(19) $$Precision=\frac{TP}{TP+FP}$$

(20) $$Recall=\frac{TP}{TP+FN}$$

(21) $$F1=\frac{2\times Precision\times Recall}{Precision+Recall}$$

AUC is used to evaluate the overall discriminative ability of the model under different decision thresholds.

As shown in Table 3, SPIMA-Net achieves the best accuracy, precision, and F1-score, reaching 98.14%, 97.22%, and 96.55%, respectively, with an AUC of 0.9983. Compared with the image-only model, the accuracy and F1-score of SPIMA-Net are approximately 5.57 and 10.44 percentage points higher, respectively, indicating that final-state images alone are insufficient to fully identify quality abnormalities caused by span deviations. Compared with the span-only model, SPIMA-Net improves accuracy, precision, and F1-score by approximately 1.86, 8.33, and 3.04 percentage points, respectively, suggesting that span priors are important for identifying nonconforming samples, but geometric information alone cannot fully capture local contour and morphological abnormalities. Compared with the direct-fusion model, SPIMA-Net improves accuracy and F1-score by approximately 3.34 and 6.27 percentage points, respectively, demonstrating that the span-prior-guided gating mechanism provides stronger fusion-based discriminative capability than simple feature concatenation. Compared with SE-fusion and CBAM-fusion, SPIMA-Net further improves accuracy by 0.74 and 0.37 percentage points and F1-score by 1.38 and 0.66 percentage points, respectively. In particular, SPIMA-Net achieves the same recall as CBAM-fusion while obtaining higher precision and fewer misclassifications, indicating a more balanced trade-off between nonconforming-sample recognition and false-alarm control. This result also indicates that simply adding image information to span information does not necessarily improve the classification performance. Since span deviation is a strong discriminative cue in this task, naïve feature concatenation may introduce irrelevant visual variations, such as local appearance changes, imaging noise, or background interference, thereby weakening the dominant role of span prior information. This further supports the necessity of the proposed span-prior-guided gating mechanism for adaptive multimodal fusion.

To further evaluate the stability of model performance under different data partitions, repeated stratified random experiments were conducted. As shown in Table 4, SPIMA-Net maintained stable performance across different random splits, indicating that the reported results were not dependent on a single train–test partition.

To further evaluate the statistical reliability of the observed performance differences, a statistical significance analysis was conducted based on three repeated stratified random experiments with different random seeds. The nonconforming-class F1-score and AUC were used as evaluation metrics. SPIMA-Net was compared with each compared model, and the mean difference, Bootstrap 95% confidence interval, paired *t*-test *p*-value, and Wilcoxon signed-rank test *p*-value were calculated. The mean difference was defined as the performance of SPIMA-Net minus that of the compared model.

As shown in Table 5, SPIMA-Net achieved positive mean differences over most compared models. For the two fusion baselines, SPIMA-Net improved the nonconforming-class F1-score and AUC by 0.0032 and 0.0017 over SE-fusion, respectively. Compared with CBAM-fusion, SPIMA-Net improved the nonconforming-class F1-score by 0.0116, with a Bootstrap 95% confidence interval of [0.0075, 0.0139] and a paired *t*-test *p*-value of 0.0291. The AUC was improved by 0.0029, with a Bootstrap 95% confidence interval of [0.0013, 0.0046]. These results provide additional statistical evidence for the performance improvement of SPIMA-Net over the compared fusion baselines.

**Figure 4:**
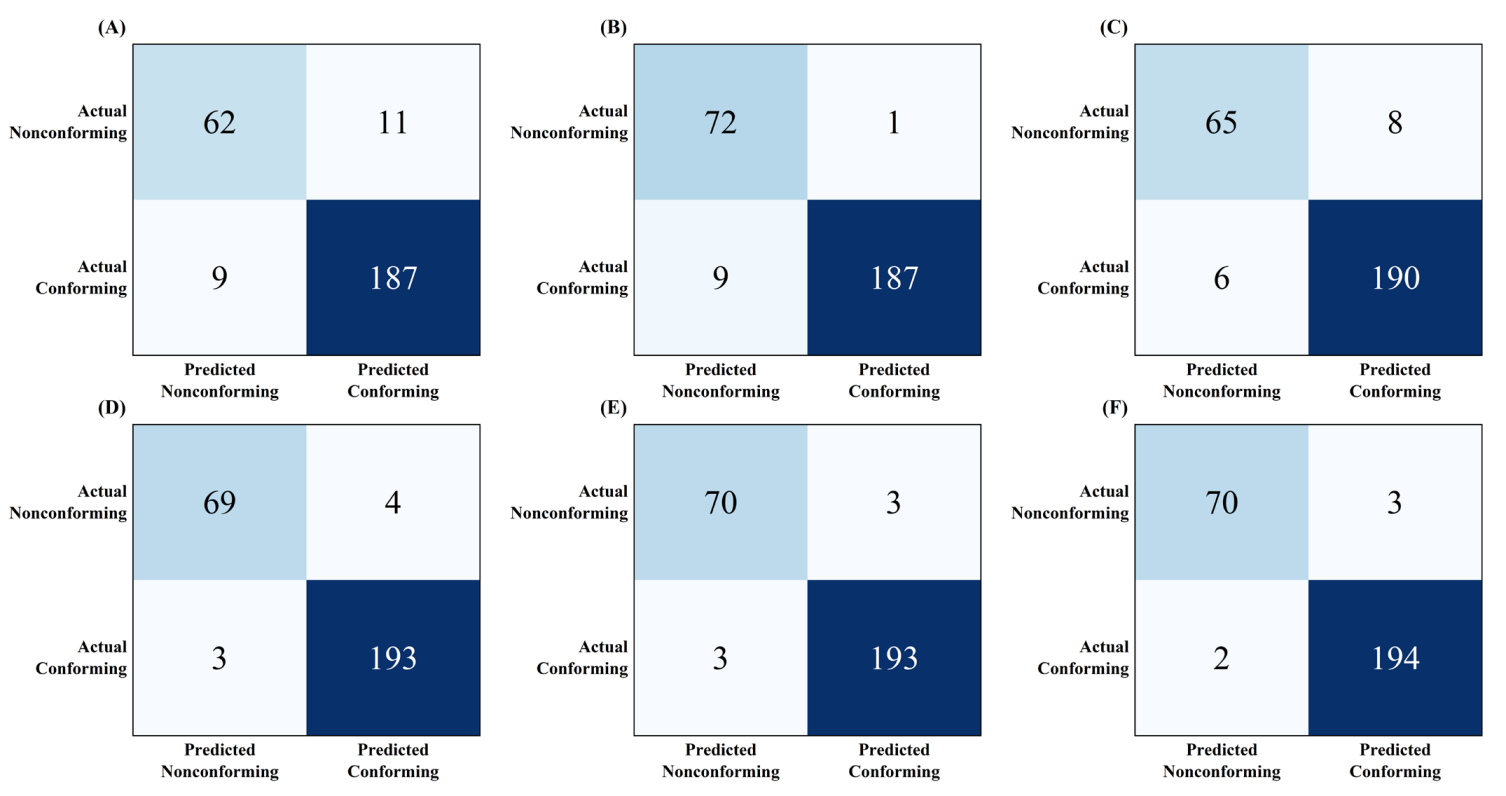
Confusion matrices of the six compared models: (**A**) Image-only; (**B**) Span-only; (**C**) Direct-fusion; (**D**) SE-fusion; (**E**) CBAM-fusion; (**F**) SPIMA-Net.

To further analyze the misclassification characteristics of different models, confusion matrices were constructed by treating nonconforming samples as the positive class. The confusion matrix statistics of the six compared models are presented in Table 6 and Figure 4.

As shown in Figure 4A, the image-only model produces 20 misclassifications, including FN = 11 and FP = 9, indicating that final-state images alone remain insufficient for identifying some span-related abnormal samples. In Figure 4B, the span-only model reduces the total number of misclassifications to 10, with FN = 1 and FP = 9. This suggests that span priors are highly sensitive to nonconforming samples, but geometric information alone tends to generate false alarms for boundary conforming samples. As shown in Figure 4C, the direct-fusion model yields 14 misclassifications, including FN = 8 and FP = 6. Although it improves upon the image-only model, simple feature concatenation does not fully exploit the complementarity between image information and span priors.

After introducing attention-based fusion mechanisms, the classification balance is further improved. As shown in Figure 4D, SE-fusion produces seven misclassifications, with FN = 4 and FP = 3. In Figure 4E, CBAM-fusion further reduces the total number of misclassifications to six, with FN = 3 and FP = 3, showing competitive performance close to SPIMA-Net. By comparison, Figure 4F shows that SPIMA-Net achieves the lowest number of misclassifications, with FN = 3 and FP = 2. More specifically, SPIMA-Net maintains a low missed-detection rate for nonconforming samples while further reducing false alarms for conforming samples compared with CBAM-fusion. This indicates that the span-prior-guided gating mechanism can more effectively use mechanical geometric information to modulate visual features, thereby strengthening the discriminative capability of final-state quality inspection for hairpin windings.

Taken together, Table 3, Table 4, Table 5 and Table 6 and Figure 4 indicate that final-state images and span priors are clearly complementary. On this basis, the introduction of the span-prior-guided gating mechanism further improves overall performance, nonconforming-sample recognition, and misclassification control, thereby validating the effectiveness of the SPIMA-Net architecture.

### 3.3. Stability Analysis Across Different Model Types

**Figure 5:**
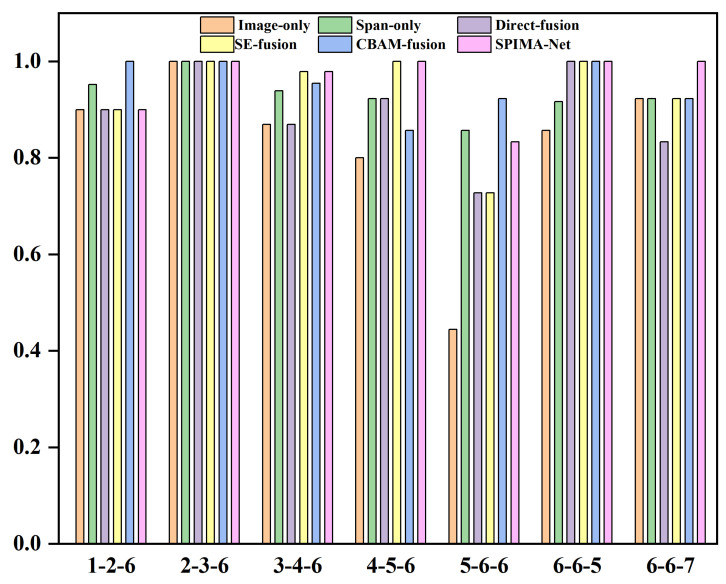
Comparison of nonconforming-class F1-scores among the six models across different model types.

In this study, model-type labels such as 1-2-6, 2-3-6, and 6-6-7 denote internal product identifiers for different stamped hairpin winding specifications on the production line. To further evaluate the stability of the proposed method across different model types, the F1-scores for the nonconforming class are calculated for six models on the test samples from seven model types, as shown in Figure 5.

Overall, SPIMA-Net maintains high recognition performance for most model types, with an overall test-set F1-score of 0.9655. Specifically, the F1-scores for the nonconforming class reach 1.0000 for the 2-3-6, 4-5-6, 6-6-5, and 6-6-7 model types, while those for the 1-2-6 and 3-4-6 model types are 0.9000 and 0.9787, respectively. The SE-fusion model achieves identical F1-scores to SPIMA-Net on five model types but drops markedly on the 5-6-6 (0.7273) and 6-6-7 (0.9231) model types. The CBAM-fusion model reaches 1.0000 on the 1-2-6, 2-3-6, and 6-6-5 model types, and achieves 0.9231 on the 5-6-6 and 6-6-7 model types, yet it exhibits lower F1-scores on the 3-4-6 (0.9545) and 4-5-6 (0.8571) model types. These results indicate that the attention-based fusion models generally improve model-type-level stability, while SPIMA-Net achieves a more balanced performance across different model types.

For the 5-6-6 model type, SPIMA-Net achieves an F1-score of 0.8333, which is substantially higher than that of the image-only model (0.4444), the direct-fusion model (0.7273), and the SE-fusion model (0.7273), and close to that of the span-only model (0.8571). The CBAM-fusion model achieves a higher F1-score of 0.9231 on this model type. This result indicates that SPIMA-Net is not superior for every individual model type, but it maintains a more balanced overall performance across different specifications. For samples with more complex local morphological differences and closer geometric boundaries, image information alone is more susceptible to interference from visually similar appearances, whereas span priors can provide effective mechanical geometric constraints for quality classification. Figure 5 shows that the span-prior-guided mechanism helps maintain classification stability across different model types.

### 3.4. Risk Scoring and Interpretability Analysis

To further analyze the discriminative capability of model output probabilities, the representational effectiveness of risk scores, and the basis for model decisions, the proposed model and the compared baselines are evaluated from four aspects: ROC curves, risk-score distributions, Grad-CAM visualization, and span-branch feature importance.

**Figure 6:**
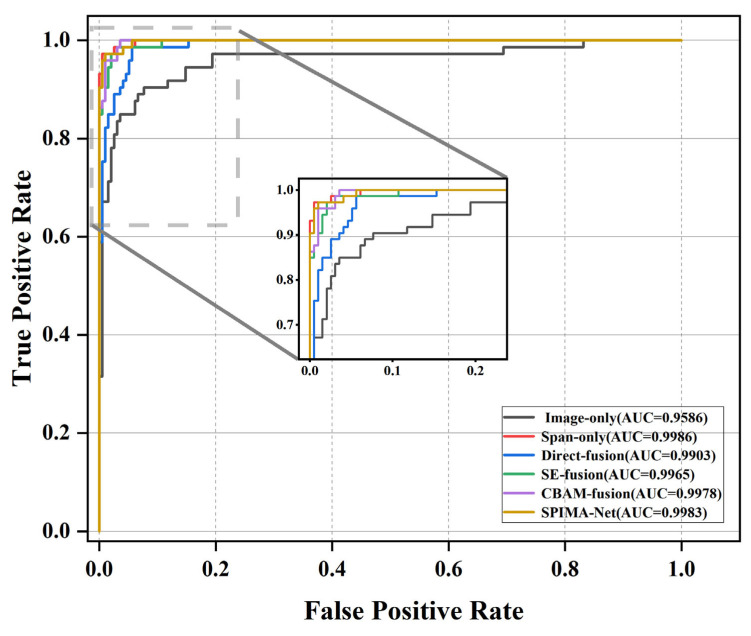
Comparison of ROC curves among the six compared models.

Figure 6 presents the ROC curves of the six compared models on the test set. All six models exhibit discriminative capability to different degrees. SPIMA-Net achieves an AUC of 0.9983, indicating strong capability to distinguish conforming and nonconforming samples. Although the span-only model obtains a slightly higher AUC of 0.9986, the confusion matrix results reported above show that it produces more false positives under the practical decision threshold. Compared with CBAM-fusion, SPIMA-Net achieves a slightly higher AUC and fewer misclassifications. These results indicate that SPIMA-Net maintains strong probability-level discrimination while achieving better misclassification control under the practical decision threshold.

**Figure 7:**
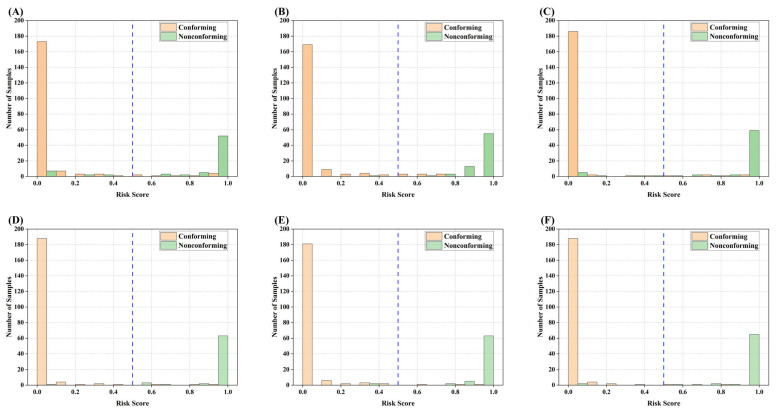
Risk-score distributions of the six compared models: (**A**) Image-only; (**B**) Span-only; (**C**) Direct-fusion; (**D**) SE-fusion; (**E**) CBAM-fusion; (**F**) SPIMA-Net.

Figure 7 shows the distributions of risk scores for the six compared models on the test set, with 0.5 used as the risk decision threshold. As shown in Figure 7A, for the image-only model, 187 out of 196 conforming samples fall into the low-risk region, and 62 out of 73 nonconforming samples fall into the high-risk region, although some overlap still exists. Figure 7B shows that the span-only model assigns 72 nonconforming samples to the high-risk region, but at the same time classifies nine conforming samples as high-risk, indicating that although span information alone is highly sensitive to nonconforming samples, it still tends to assign excessively high risk scores to some boundary samples. In Figure 7C, the direct-fusion model shows an improved risk distribution compared with the image-only model, with 190 conforming samples located in the low-risk region and 65 nonconforming samples located in the high-risk region, but the two classes are still not fully separated. The attention-based fusion variants also exhibit a more compact and better separated risk-score distribution. As shown in Figure 7D, SE-fusion assigns 69 nonconforming samples to the high-risk region while keeping 193 conforming samples in the low-risk region. Similarly, Figure 7E shows that CBAM-fusion assigns 70 nonconforming samples to the high-risk region and retains 193 conforming samples in the low-risk region. These results indicate that SE-fusion and CBAM-fusion improve the risk separation between conforming and nonconforming samples, although a small number of boundary samples still remain near or across the decision threshold. By comparison, the distribution of SPIMA-Net in Figure 7F is slightly more concentrated, with 194 conforming samples located in the low-risk region and 70 nonconforming samples located in the high-risk region, with only a few samples crossing the threshold. This indicates that the span-prior-guided gating mechanism can further improve the discrimination capability and stability of risk scoring.

**Figure 8:**
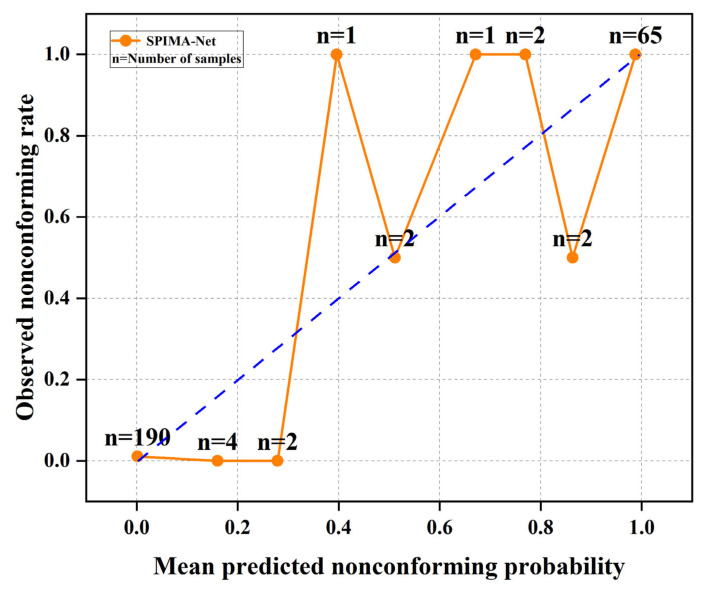
Reliability diagram of SPIMA-Net.

Based on the risk-score distributions of the compared models shown in Figure 7, SPIMA-Net exhibits clear risk discrimination between conforming and nonconforming samples. On this basis, the calibration behavior of its predicted risk scores was further evaluated using the ECE and Brier score results in Table 4 together with the reliability diagram in Figure 8. As reported in Table 4, SPIMA-Net achieved the lowest ECE of 0.0200 ± 0.0114 and the lowest Brier score of 0.0147 ± 0.0090 among the compared models, indicating favorable calibration performance. Figure 8 further shows a clear calibration tendency of SPIMA-Net, particularly in the low-risk and high-risk regions. The local fluctuations in the observed nonconforming rate within the middle-probability intervals may be influenced by the uneven sample distribution across these bins. Therefore, the predicted risk score of SPIMA-Net can provide useful relative risk information for final-state quality inspection, while it should not be interpreted as a fully calibrated absolute probability under all deployment conditions.

**Figure 9:**
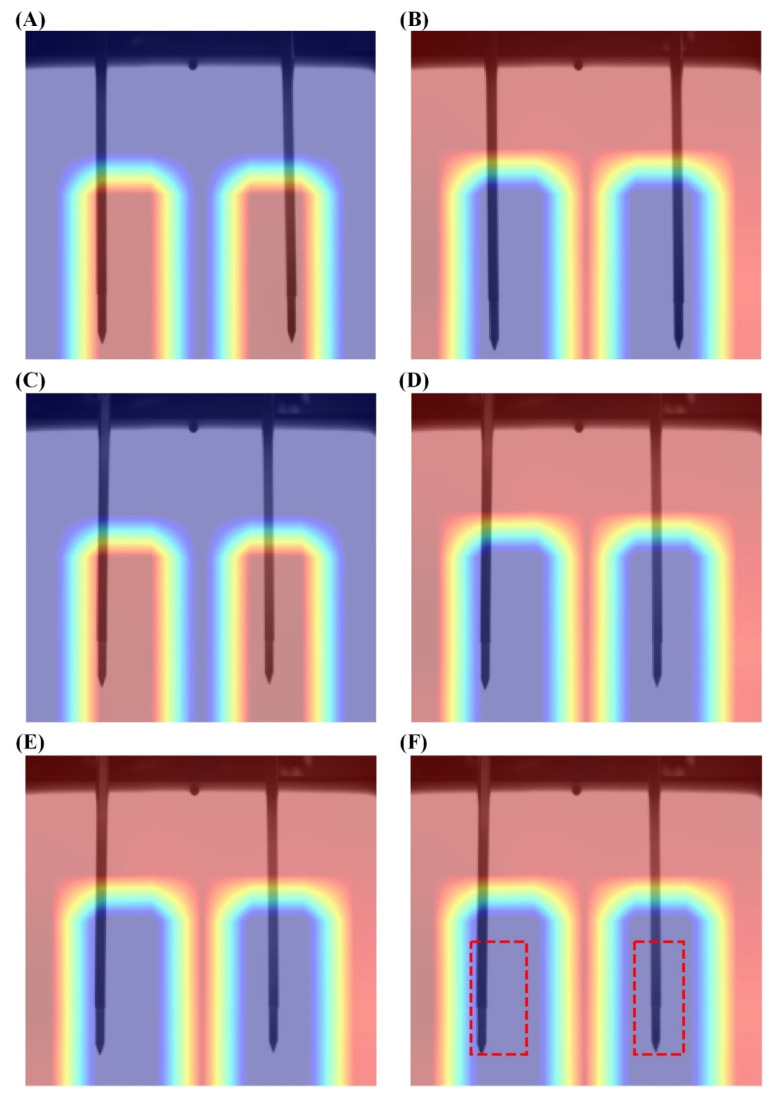
Grad-CAM visualization and terminal ROI comparison of SPIMA-Net: (**A**) nonconforming sample of model type 3-4-6; (**B**) conforming sample of model type 3-4-6; (**C**) nonconforming sample of model type 6-6-5; (**D**) conforming sample of model type 6-6-5; (**E**) Grad-CAM visualization of a conforming sample of model type 6-6-5; (**F**) terminal ROI comparison of the sample in (**E**).

Figure 9 presents representative Grad-CAM visualization results of SPIMA-Net. Specifically, Figure 9A and Figure 9B show the Grad-CAM results of a nonconforming and a conforming sample of model type 3-4-6, respectively. Figure 9C and Figure 9D show the Grad-CAM results of a nonconforming and a conforming sample of model type 6-6-5, respectively. Figure 9E shows the Grad-CAM result of a conforming sample of model type 6-6-5, and Figure 9F further provides the comparison between this sample and the terminal span-based quality assessment ROI. Warmer colors indicate stronger Grad-CAM responses, whereas cooler colors indicate weaker responses. Background color differences outside the activated regions are used only for visual distinction and are not involved in the evaluation. The red dashed boxes indicate the predefined ROI regions related to terminal span-based quality assessment. It can be observed that the high-response regions of SPIMA-Net are mainly concentrated around the span opening and the terminal pins on both sides, rather than being distributed over irrelevant background regions. This result indicates that, when distinguishing between conforming and nonconforming samples, the model can effectively focus on local morphological features associated with final-state span quality. The highlighted regions are therefore consistent with the key geometric regions considered in final-state quality inspection of hairpin windings, further supporting the interpretability of the proposed method.

Based on the Grad-CAM visualization results in Figure 9, an ROI-based activation analysis was performed to quantify the image-side interpretability of SPIMA-Net. The terminal-span quality-assessment regions were defined as ROIs. The ROI activation ratio was calculated as the Grad-CAM activation sum within the ROI divided by the total Grad-CAM activation sum of the whole image, and the ROI enrichment factor was calculated as the ROI activation ratio divided by the ROI area ratio. Therefore, the ROI activation ratio reflects the proportion of model response located in the ROI, while the ROI enrichment factor reflects whether the response is concentrated in the ROI relative to its area.

As shown in Table 7, the representative nonconforming samples show substantially higher ROI activation ratios and ROI enrichment factors than the conforming samples. Based on the sample results in Table 7, the mean ROI activation ratio was 0.3550 for nonconforming samples and 0.0033 for conforming samples, while the mean ROI enrichment factor was 3.0091 for nonconforming samples and 0.0279 for conforming samples. These results quantitatively indicate that SPIMA-Net assigns stronger Grad-CAM responses to terminal-span-related ROI regions when identifying nonconforming final states.

**Figure 10:**
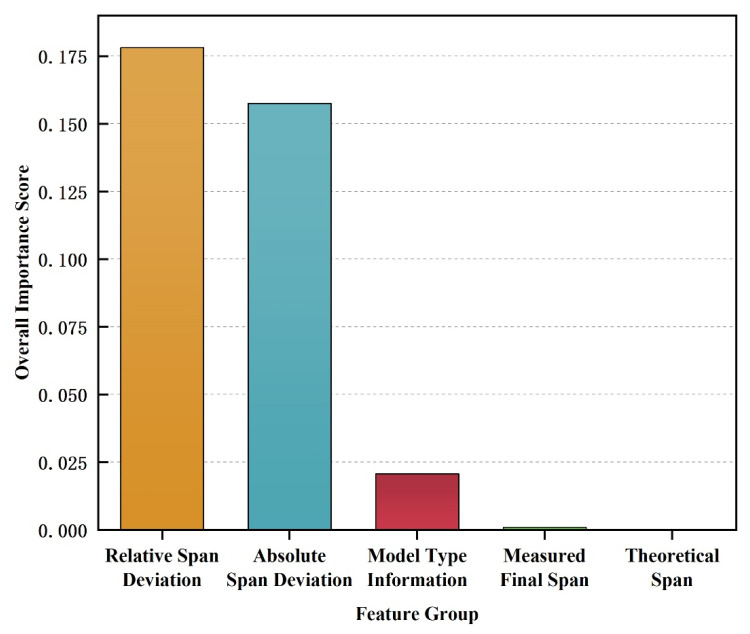
Feature importance of the span branch in SPIMA-Net.

The feature-group importance results of the span branch are presented in Table 8 and Figure 10. To provide a unified evaluation of the contribution of different span prior variables to model discrimination, a permutation-based feature-group importance analysis was conducted on the test set. Specifically, each span prior feature group was permuted, and the decreases in F1-score and AUC before and after perturbation were calculated. The mean F1 decrease and mean AUC decrease were then combined to obtain the Overall Importance Score. Since these span prior variables jointly describe the geometric deviation between the measured final span and the theoretical target, their importance scores are analyzed as feature-group-level sensitivity rather than as isolated single-variable effects. This treatment is consistent with the purpose of the analysis, which is to identify which type of span-related information has a greater influence on the classification performance of SPIMA-Net.

As shown in Table 8 and Figure 10, relative span deviation and absolute span deviation achieve the highest Overall Importance Scores, reaching 0.1782 and 0.1576, respectively, which are substantially higher than those of the other features. This indicates that span-deviation-related information is the main mechanical geometric factor affecting nonconforming classification. The Overall Importance Score of model-type information is 0.0207, suggesting that differences in geometric boundaries among model types also influence model decisions. By contrast, the importance scores of the measured final span and theoretical span are relatively low, indicating that the model pays more attention to the deviation of the measured dimension from the theoretical target than to a single raw dimensional value.

Taken together, the above results show that the advantage of SPIMA-Net does not simply arise from the addition of input information but from the effective guidance of visual features by span priors. The proposed method achieves a balanced performance in classification accuracy, risk discrimination capability, and engineering interpretability, demonstrating its effectiveness for final-state quality inspection of hairpin windings.

## 4. Discussion

Existing studies on industrial visual inspection and multimodal fusion have shown that image representation and multisource information integration can improve complex quality inspection performance. However, for final-state quality inspection of hairpin windings with explicit geometric decision criteria, relying only on visual information or using conventional feature concatenation remains insufficient to capture the relationships among final-state morphology, span deviation, and quality status. The results of this study indicate that using span geometric priors as guiding signals for visual feature modulation is more effective for this type of mechanically formed quality inspection task.

This finding verifies the core hypothesis of this study, namely that span prior guidance can enhance the consistency between visual representation and quality classification. Span information provides evidence of key dimensional deviations, while final-state images supplement discriminative cues such as end profiles, opening regions, and local abnormal morphologies. The advantage of SPIMA-Net does not arise from simple stacking of input modalities. Instead, it relies on a collaborative decision mechanism. In this mechanism, geometric priors guide the decision direction, while visual features provide local evidence. In addition, Grad-CAM visualization and span feature importance analysis show that the model’s attention regions and major decision factors are consistent with engineering inspection knowledge. This indicates that the proposed method not only improves inspection performance but also enhances the interpretability of the decision process. The proposed idea may provide a useful reference for other mechanically formed quality inspection tasks with explicit dimensional constraints, and its applicability can be further extended by incorporating richer three-dimensional geometric features.

In this study, the classification performance and interpretability of SPIMA-Net were evaluated using an industrial dataset covering seven hairpin winding model types with different geometric characteristics. All samples were collected from the same manufacturing environment, production line, production batch, and fixed imaging setup. Variations in independent production batches, different production lines, illumination conditions, camera positions, and backgrounds may lead to distribution shifts in both image features and span prior features, thereby affecting model performance, risk-score reliability, and deployment adaptability. Therefore, model evaluation under cross-batch, cross-line, and cross-imaging conditions will be an important direction of future work to further improve the robustness and industrial applicability of the proposed method.

## 5. Conclusions

This study proposes a span-prior-guided explainable multimodal neural network method and develops SPIMA-Net as its core model. The proposed method combines final-state images with span-related geometric priors and employs a span-prior-guided gating mechanism to modulate visual features, thereby achieving collaborative fusion between image representations and mechanical geometric information for final-state quality inspection and risk scoring of three-dimensional stamped hairpin windings.

Experimental results show that SPIMA-Net achieves an accuracy of 98.14%, a precision of 97.22%, a recall of 95.89%, an F1-score of 96.55%, and an AUC of 0.9983 on the test set. Compared with the image-only, span-only, direct-fusion, SE-fusion, and CBAM-fusion models, the F1-score for the nonconforming class is improved by 10.44, 3.04, 6.27, 1.38, and 0.66 percentage points, respectively, while the total number of misclassifications decreases to five. Although CBAM-fusion shows competitive performance close to SPIMA-Net in some evaluations, SPIMA-Net achieves more balanced overall performance in terms of classification accuracy, nonconforming-sample recognition, and misclassification control. Further analysis shows that SPIMA-Net maintains stable classification performance across different model types, with an overall test-set F1-score of 0.9655. Risk-score distributions, reliability analysis, Grad-CAM visualization, and span-feature importance analysis further verify the effectiveness of the proposed method in risk representation and engineering interpretability.

Although SPIMA-Net achieved high classification performance and interpretable decision evidence on the investigated industrial dataset covering seven hairpin winding model types, further model evaluation under independent production batches, different production lines, and different imaging conditions will be investigated in future work. For production deployment, an input-checking module can be introduced before SPIMA-Net inference to identify missing model-type information, abnormal span measurements, or corrupted images. When model-type or span-related information is unavailable, the system can switch to an image-only backup inference mode and flag the sample for manual review; when the image input is corrupted, image reacquisition should be triggered before quality judgment. Future work will combine three-dimensional geometric enhancement, multimodal representation learning, and cross-scenario adaptation mechanisms to further improve the robustness, extensibility, and long-term deployment potential of the proposed framework.

## Figures and Tables

**Table 1:** Structural components and input information of different models.

Model Name	Image Branch	Span Branch	Fusion Strategy	Output
Image-only	ResNet18	No	No	Class probability
Span-only	No	MLP	No	Class probability
Direct-fusion	ResNet18	MLP	Direct concatenation	Class probability
SE-fusion	ResNet18	MLP	SE-based fusion	Class probability
CBAM-fusion	ResNet18	MLP	CBAM-based fusion	Class probability
SPIMA-Net	ResNet18	MLP	Prior-assisted gating + concatenation	Class probability + risk score

**Table 2:** Dataset composition and partition statistics.

Dataset	Number of Conforming Samples	Number of Nonconforming Samples	Total Number of Samples
Overall Samples	982	361	1343
Training Set (80%)	786	288	1074
Test Set (20%)	196	73	269

**Table 3:** Overall performance comparison of different models on the test set.

Model Name	Accuracy%	Precision%	Recall%	F1%	AUC
Image-only	92.57	87.32	84.93	86.11	0.9586
Span-only	96.28	88.89	98.63	93.51	0.9986
Direct-fusion	94.80	91.55	89.04	90.28	0.9903
SE-fusion	97.40	95.83	94.52	95.17	0.9965
CBAM-fusion	97.77	95.89	95.89	95.89	0.9978
SPIMA-Net	98.14	97.22	95.89	96.55	0.9983

**Table 4:** Repeated random experiment results under different data partitions.

Model	Accuracy (%)	Precision (%)	Recall (%)	F1-Score (%)	AUC	ECE	Brier Score
Image-only	94.55 ± 1.20	89.96 ± 2.04	89.95 ± 2.76	89.95 ± 2.25	0.9767 ± 0.0132	0.0397 ± 0.0080	0.0421 ± 0.0077
Span-only	97.65 ± 1.41	92.92 ± 4.95	99.09 ± 0.79	95.85 ± 2.43	0.9991 ± 0.0008	0.0462 ± 0.0068	0.0175 ± 0.0068
Direct-fusion	96.78 ± 0.86	92.16 ± 2.17	96.35 ± 0.79	94.20 ± 1.51	0.9952 ± 0.0035	0.0239 ± 0.0067	0.0247 ± 0.0090
SE-fusion	97.89 ± 0.86	96.42 ± 3.30	95.89 ± 1.37	96.13 ± 1.54	0.9972 ± 0.0021	0.0214 ± 0.0026	0.0181 ± 0.0051
CBAM-fusion	97.40 ± 1.34	94.30 ± 3.99	96.35 ± 0.79	95.29 ± 2.29	0.9960 ± 0.0021	0.0278 ± 0.0108	0.0220 ± 0.0114
SPIMA-Net	98.02 ± 1.31	94.37 ± 3.16	98.63 ± 1.37	96.44 ± 2.31	0.9989 ± 0.0006	0.0200 ± 0.0114	0.0147 ± 0.0090

**Table 5:** Statistical significance analysis between SPIMA-Net and compared models.

Compared Model	Metric	Mean Difference	Bootstrap 95% CI	Paired *t*-Test *p*-Value	Wilcoxon *p*-Value
Image-only model	F1-score	0.0650	[0.0515, 0.0821]	0.0187	0.2500
Span-only model	F1-score	0.0059	[−0.0077, 0.0253]	0.6108	0.7500
Direct-fusion model	F1-score	0.0224	[0.0071, 0.0331]	0.1044	0.2500
SE-fusion model	F1-score	0.0032	[−0.0126, 0.0147]	0.7343	0.7500
CBAM-fusion model	F1-score	0.0116	[0.0075, 0.0139]	0.0291	0.2500
Image-only model	AUC	0.0221	[0.0073, 0.0303]	0.0962	0.2500
Span-only model	AUC	−0.0002	[−0.0013, 0.0009]	0.7697	0.7500
Direct-fusion model	AUC	0.0037	[0.0007, 0.0064]	0.1580	0.2500
SE-fusion model	AUC	0.0017	[−0.0002, 0.0028]	0.2171	0.5000
CBAM-fusion model	AUC	0.0029	[0.0013, 0.0046]	0.0936	0.2500

**Table 6:** Confusion matrix statistics of the six compared models.

Model Name	TP	FN	FP	TN	Number Predicted as Nonconforming	Number Predicted as Conforming	Total Number of Misclassifications
Image-only	62	11	9	187	71	198	20
Span-only	72	1	9	187	81	188	10
Direct-fusion	65	8	6	190	71	198	14
SE-fusion	69	4	3	193	72	197	7
CBAM-fusion	70	3	3	193	73	196	6
SPIMA-Net	70	3	2	194	72	197	5

**Table 7:** Quantitative Grad-CAM ROI activation analysis of SPIMA-Net.

Hairpin Type	Sample Type	Distance	ROI Activation Ratio	ROI Enrichment Factor
6-6-5	Nonconforming	57.34	0.3551	3.0095
6-6-5	Nonconforming	57.34	0.3551	3.0095
3-4-6	Nonconforming	65.23	0.3550	3.0086
3-4-6	Nonconforming	65.32	0.3550	3.0086
6-6-5	Conforming	58.96	0.0033	0.0279
6-6-5	Conforming	58.99	0.0033	0.0279
3-4-6	Conforming	63.93	0.0033	0.0279
3-4-6	Conforming	64.41	0.0033	0.0279

**Table 8:** Ranking of feature importance in the span branch of SPIMA-Net.

Rank	Feature Name	Mean F1 Decrease	Mean AUC Decrease	Overall Importance Score
1	Relative Span Deviation	0.157395	0.020816	0.178211
2	Absolute Span Deviation	0.141929	0.015667	0.157596
3	Model-Type Information	0.018320	0.002411	0.020732
4	Measured Final Span	0.000840	0.000198	0.001038
5	Theoretical Span	−0.001337	0.000262	0.000262

## Data Availability

The data supporting the findings of this study are not publicly available due to confidentiality requirements of the industrial application scenario.
